# A Cross-Sectional and Longitudinal Study of Novel Metaphor and Metonymy Comprehension in Children, Adolescents, and Adults With Autism Spectrum Disorder

**DOI:** 10.3389/fpsyg.2018.00945

**Published:** 2018-06-11

**Authors:** Jo Van Herwegen, Gabriella Rundblad

**Affiliations:** ^1^Department of Psychology, Kingston University London, London, United Kingdom; ^2^School of Education, Communication and Society, King’s College London, London, United Kingdom

**Keywords:** novel metaphor, novel metonymy, ASD, longitudinal, cross-sectional, development

## Abstract

Previous studies have shown that comprehension of figurative language is impaired in individuals with autism spectrum disorder (ASD). However, most studies have focused on lexicalized expressions and have only examined performance at one particular point in time, without examining how performance changes over development. The current study examined the comprehension of novel metaphor and metonymy in individuals with ASDs from a large age range, using both a cross-sectional (Experiment 1) and longitudinal design (Experiment 2). Performance in the ASD group was lower compared to typically developing (TD) controls, across all ages. Importantly, the results from Experiments 1 and 2 showed that, although chronological age was not a good predictor for performance of either novel metaphor or metonymy in the cross-sectional design, performance improved when longitudinal data was considered. Correlations between vocabulary knowledge, visuo-spatial abilities and figurative language comprehension abilities were also explored.

## Introduction

Autism spectrum disorder (ASD) is a neurodevelopmental disorder that is diagnosed on the basis of a dyad of behavioral impairments in the areas of social interaction and communication, as well as restricted and repetitive activities or interests (DSM V, American Psychiatric Association, 2013). ASD is characterized by a specific language and communicative profile which includes impaired pragmatic communication or the ability to use language in social contexts (Tager-Flusberg, 1996). One particular language problem that has been frequently reported is the comprehension of figurative expressions, in that individuals with ASD often interpret figurative language literally (see Kalandadze et al., 2016, for a meta-analysis). For example, Dennis et al. (2001) examined performance in eight high-functioning children with ASD on an inference task and a metaphor task. They found that ASD children were less able to understand metaphors and idioms than typically developing (TD) peers, even though they had no problems understanding that a word can have different meanings. Likewise, MacKay and Shaw (2004) investigated comprehension of a range of figurative expressions, including metonymy, hyperbole, irony, etc. Their results showed that children with ASD understood most expressions literally and that they struggled to understand the intent of figurative expressions. However, recent studies (Kasirer and Mashal, 2014, 2016; Zheng et al., 2015) did not find that participants with ASD were impaired on metaphor comprehension compared to TD peers. One reason why previous studies have found contradictory results is that they examined performance of participants from different age ranges at one particular point in time, either focusing on very young children (Rundblad and Annaz, 2010b) or only on adolescents and adults (Kasirer and Mashal, 2014). Yet, research in other developmental disorders, such as Williams syndrome, have shown that comprehension abilities change over development (Van Herwegen et al., 2013). The current study addresses this shortcoming by examining the development of metaphor and metonymy comprehension in ASD and how comprehension abilities improve with time using both a cross-sectional and longitudinal design.

In addition, many studies have failed to distinguish between different types of expressions (e.g., similes and conceptual metaphors), even though different types of figurative expressions have been shown to vary in level of comprehension difficulty (Rundblad and Annaz, 2010a; Rundblad, 2017). Metaphors are figurative expressions in which two concepts from different conceptual domains, namely the target that is being referred to and the vehicle that is used in the reference, are linked based upon the fact that they share some common ground. In the expression “My teacher is a dragon,” the teacher is referred to in terms of a mystical figure, highlighting that both the teacher and a dragon are fierce. Metonyms are another type of figurative expressions that are commonly used in daily conversations. In contrast to metaphors, the target and vehicle in metonyms are linked based upon continuity and thus they belong to the same conceptual domain. For example, in the expression “The sax has flu today,” the instrument is used to refer to the person playing the saxophone. Recent research in TD children has shown that comprehension of metonyms starts at an earlier age compared to metaphors and thus it can be argued that metonyms are easier compared to metaphors (Rundblad and Annaz, 2010a). However, a recent study examining metaphor and metonymy in Chinese children with ASD found the opposite pattern (Zheng et al., 2015).

The use of a variety of methodologies (see Melogno et al., 2012, for a review) is yet another contributor to seemingly conflicting results; for example, most studies in ASD have focused on the comprehension of metaphors (see Kalandadze et al., 2016), and many studies have favored tasks requiring participants to verbalize their understanding (see Rundblad, 2017, for a discussion). Another methodological issue in previous studies investigating figurative language comprehension in ASD has been the preference to test lexicalized figurative expressions (Happé, 1993; Norbury, 2005; Rundblad and Annaz, 2010b). For lexicalized figurative expressions, the meaning might have been encountered so many times before so that the child can select the correct meaning directly from his or her mental lexicon (Bowdle and Gentner, 2005); thus, it might not be surprising that comprehension of lexicalized expressions is related to receptive vocabulary (Rundblad and Annaz, 2010a) and language abilities (see Gernsbacher and Pripas-Kapit, 2012, for a discussion). However, an alternative interpretation as to why participants with ASD fail to understand figurative expressions, such as metaphor and metonyms, is that they fail to establish a common ground or relationship between the target and the vehicle (Gold and Faust, 2012) or discern a pattern across multiple target-vehicle pairs thereby being unable to integrate that semantic information (Coulson and Van Petten, 2002). Yet, it is currently unclear for which lexicalized expressions participants can directly access the meaning in their mental lexicon and for which a meaning has to be created on-line (Bowdle and Gentner, 2005). Novel metaphors and metonyms differ from lexicalized ones in that their meaning has never (or rarely) been encountered before. Hence, novel metaphors and metonyms always require that a new meaning is created (sense creation) by establishing the common ground between the target and the vehicle. Looking at novel expressions that children have never encountered before (e.g., “her hair is spaghetti” or “the mop is coming tomorrow”), will establish how the development of metaphor comprehension compares to the development of metonymy comprehension, as well as which cognitive abilities (i.e., verbal and non-verbal abilities) can predict metaphor and metonymy comprehension, if any. Thus far, very few studies have investigated novel metaphor and metonymy comprehension in ASD (see Rundblad, 2017, for a review).

A study by Olofson et al. (2014) examined the comprehension of novel primary conceptual metaphors, which are the building blocks of metaphoric competence, in youth with ASD, and found that, although the participants with ASD performed above chance and were able to understand the expressions, their comprehension abilities were lower than the TD controls. However, not all studies have found evidence that comprehension of novel metaphor is impaired in ASD. Mashal and Kasirer (2011) found that, although adolescents with ASD aged 12 to 15 had lower performance on lexicalized metaphors compared to TD controls in a multiple-choice task, there were no difference for novel metaphors and unrelated word pairs. Hermann et al. (2013) found that processing of novel metaphors in 20 adults with Asperger syndrome (aged 22–68) was similar to performance of age-matched TD individuals. A study by Zheng et al. (2015) has shown that comprehension of novel metaphors and metonyms in Chinese children with ASD did not differ to performance in the control group, in contrast to comprehension of lexicalized expressions. A recent study by Kasirer and Mashal (2016) showed that children with ASD had problems with lexicalized metaphor generation, but that the ability to understand novel metaphors was not impaired. Although there is indication that novel metaphor comprehension is less impaired than lexicalized metaphor comprehension in ASD, there is no conclusive evidence yet, and in the case of novel metonymy comprehension there is a conspicuous lack of studies to date.

With regards to which cognitive abilities might impact comprehension of novel figurative language expressions, Kasirer and Mashal (2014) examined performance on novel versus lexicalized metaphors in high functioning adults with ASD compared to age matched TD peers. They found no differences in comprehension scores between the two groups, but importantly, Kasirer and Mashal found that whilst comprehension of lexicalized expressions correlated with semantic knowledge (see also Gernsbacher and Pripas-Kapit, 2012, for a discussion), comprehension of novel expressions correlated with scores on tasks that assess mental flexibility. Gold and Faust (2010) presented adult participants with and without high functioning ASD with novel and lexicalized metaphorical word-pairs. The study found that both participants with ASD and age-matched controls took longer to respond to novel metaphorical pairs in contrast to lexicalized or control pairs. In addition, there was an overall larger N400 in the ASD group in contrast to the control group, showing that participants with ASD have general difficulties with semantic processing for both lexicalized and novel metaphors. However, this N400 effect was larger for novel than for lexicalized expressions. Consequently, the roles that verbal and non-verbal abilities may play in comprehension of novel metaphors and metonyms remain to be established.

Previous studies have mainly examined performance only at one particular point in time and have matched participants with ASD to control group either based on chronological age or based on a particular cognitive ability. However, outcomes in adults do not necessarily faithfully represent abilities in younger children. In addition, matching approaches have been found to be particularly difficult for neurodevelopmental disorders and matching on a particular cognitive ability is theory dependent, in that researchers “are taking a theory-driven view on what standardized test adequately measures developmental progression in the domain that the experimental task is thought to tap” (Thomas et al., 2009, p. 338). Therefore, a truly developmental approach is needed to understand whether novel figurative comprehension in ASD is delayed or atypical (Karmiloff-Smith, 2013).

One way of studying development is to use a cross-sectional approach in which performance of participants with neurodevelopmental disorders from various ages is plotted on a trajectory against age, and this trajectory is then compared to the trajectory of a control group (Thomas et al., 2009). However, cross-sectional studies include snapshots of cognitive abilities across different age groups, and thus, individual differences within the group may affect the trajectory or mask any real changes over time across an entire group. As a result, cross-sectional studies should be followed up by longitudinal research to confirm the developmental profiles (Van Herwegen et al., 2014).

The current study is innovative in that it is the first study to investigate the development of novel metaphor and metonymy comprehension in individuals with ASD both cross-sectionally and longitudinally. The current study employed a story comprehension task, predicting that comprehension of novel metaphor and novel metonymy would be delayed in ASD, i.e., participants with ASD would perform worse in comparison to age-matched controls, but their performance would improve with age. We also examined the relationship between novel metaphor and metonymy, and verbal ability, as it has been suggested that comprehension of lexicalized figurative language in ASD is in line with their verbal abilities. In addition, we correlated figurative language performance with non-verbal abilities in order to examine whether poor figurative comprehension is related to weak mental flexibility in ASD.

## Experiment 1

In the first experiment, we examined comprehension of novel metaphor and novel metonymy cross-sectionally in a large age group including children, adolescents and adults, comparing their performance to TD participants whose chronological age fell within those of the ASD group.

### Method

#### Participants

Eighteen younger individuals (16 males and 2 females), including children and adolescents, with ASD were recruited from special needs education schools in Greater London. In addition, 16 adults with ASD (14 males and 2 females) were recruited from Kingston’s adult learning and disability service, Greater London, via the local authorities’ learning disabilities services. The average chronological age for the entire ASD group in months was 207.21 (*SD* = 126.784). All ASD participants had a clinical diagnosis for ASD established by a trained clinician (using ADOS or ADI), according to parental reports. We confirmed diagnosis using the parental questionnaire *Childhood Autism Rating Scale* for the child participants (all CARS scores were above 30; Schopler et al., 1988) and the Autism Quotient questionnaire (AQ; Baron-Cohen et al., 2001) for the older ASD participants.

Thirty-four healthy control participants (19 males and 15 females), including children and adults whose chronological age fell within the range of the ASD group, were included (mean chronological age: 209.74 months, *SD* = 128.931).

Participants in all groups were English native speakers of a similar socio-economic (as measured by mothers of the children or their own highest level of education for the adults) and ethnic background, according to the background questionnaire, and none had any hearing or vision problems. None of the participants in the control group had been diagnosed with any cognitive neurodevelopmental disorders or learning difficulties.

#### Materials

The younger participants were administered the British Picture Vocabulary Scale (BPVS: Dunn et al., 2009) to obtain vocabulary comprehension scores and the Pattern Construction task from the British Ability Scales (PC: Elliott et al., 1996) provided non-verbal ability scores. Originally, only children and adolescents were to take part in the current study, but as preliminary analysis showed that even adolescents with ASD scored well below their age-matched control group, adults were recruited to participate as well. However, as the BPVS and PC are not age-appropriate tasks for adults, adult participants were administered two sub-scales from the Wechsler Abbreviated Intelligence Scale (WAIS; Wechsler, 1999), namely the vocabulary and block design sub-scales.

In addition to the background measures, participants were administered a baseline task and the Novel Metaphor and Metonymy (M&M) task (see Van Herwegen et al., 2013 for a detailed description of the task and procedure). In order to enable participants to concentrate on the task, each story was accompanied by three black and white pictures. The baseline task was administered to ensure that all participants were able to listen to short stories between five and seven sentences long and answer questions about them. This baseline task included six unambiguous stories that were similar in structure to the novel M&M stories, but at the end the participants were asked a question about a target item explicitly mentioned at the beginning of the story. Participants who failed more than two of the six unambiguous stories in the baseline task were excluded from further analyses. All the participants in the current study were able to answer more than four stories correct on the baseline task.

**FIGURE 1:**
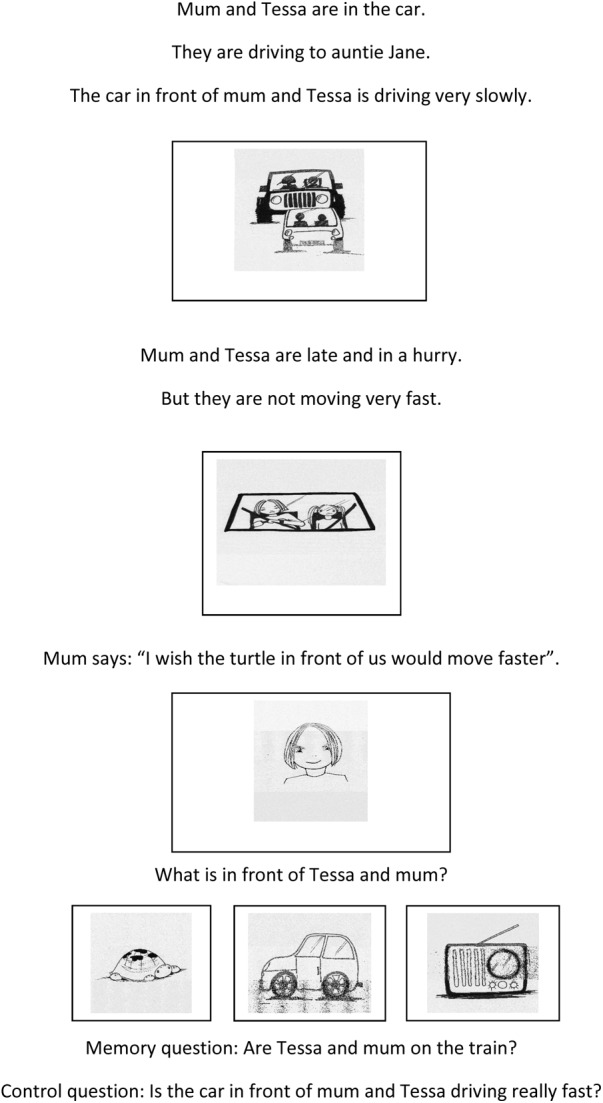
Example of a novel metaphor story.

In the Novel M&M task, participants listened to 24 stories that ended in a novel metaphor or metonym (see example in **Figure 1**).

The 24 novel expressions (12 metaphors and 12 metonyms) included six sensory metaphor expressions (i.e., the target links to the vehicle based upon the fact that they share sensory commonalities: e.g., a soft pillow was referred to as *a marshmallow*), six non-sensory metaphors (i.e., the target and vehicle share commonalities other than sensory ones: e.g., a slow car labeled *a turtle*), six object-user metonyms (i.e., they refer to a person in terms of an object; e.g., *the apron* stands for the cook), and six synecdoche metonyms (i.e., the whole target is referred to through a part of the whole; e.g., *the mustache* for the man with the mustache). The novel M&M expressions were presented to the participant in the form of short stories that were between six and seven sentences long. All the stories included story lines that are familiar to children (for example: going to school, going on a holiday, playing with toys, having dinner, etc.). At the end of each story, the participant was asked one implicit question about the meaning of the vehicle (the M&M question) and two additional questions (one memory question and a control question). The memory question asked about something mentioned in the first three sentences of the story in order to make sure that the participant had been listening from the start onwards. The control question asked explicitly about the target meaning of the vehicle. For the M&M question, three pictures were presented on the screen (a picture depicting the figurative meaning, one depicting the literal meaning, and a distracter picture). The memory and control questions were yes-no questions for which the participant had to press a green or red circle on the screen. The order of the memory and control questions was semi-randomized across the stories.

#### Procedure

Participants were instructed to listen to the short stories and were told they had to answer some questions at the end of each story. The 24 stories were presented in a semi-randomized order so that no more than two figurative expressions of the same type (either novel metaphors or novel metonyms) followed in a row. Furthermore, half of the participants were presented the stories in the reverse order, in order to limit order effects.

All standardized and experimental tasks were administered in one testing session that lasted about 1 h. Breaks were given to participants as often and as long as was required. Before testing took place, verbal assent from child and adolescent participants, as well as parental consent, and written consent from adult participants was obtained. This study was approved by the Social Sciences Faculty Research Ethics Committee at Kingston University, London (Reference No. FREC111251).

#### Scoring

A percentage score was calculated for the number of M&M questions a participant had answered correctly when the memory question and control question was answered correctly as well. This ensured that failing the M&M question was not due to attention or memory problems. All participants who answered the M&M question correctly, also answered the control question correctly, confirming their comprehension.

### Results

**FIGURE 2:**
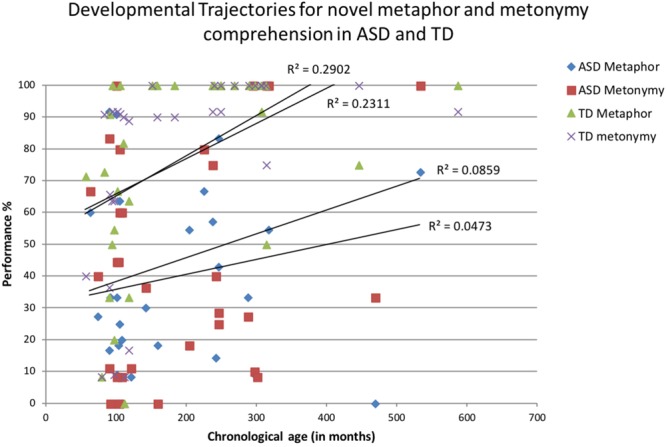
Novel metaphor and metonymy comprehension plotted against chronological age (in months) for participants with ASD and typically developing (TD) participants.

First, performance on novel M&M comprehension was plotted against CA in each group (i.e., TD and ASD), separately. Only when these cross-sectional developmental trajectories were significant, was performance compared across both groups. For novel metaphor comprehension, there was a significant relationship with CA for the TD group; *F*(1,33) = 9.920, *p* = 0.004, $\eta_{p}^{2}$ = 0.231, but not for the ASD group; *F*(1,33) = 1.589, *p* = 0.217, $\eta_{p}^{2}$ = 0.047. Similarly, novel metonymy comprehension increased significantly with increasing CA in the control group; *F*(1,33) = 13.082, *p* = 0.001, $\eta_{p}^{2}$ = 0.290, but not the ASD group; *F*(1,33) = 3.301, *p* = 0.093, $\eta_{p}^{2}$ = 0.086. Excluding participants who performed at ceiling did not change these results. As can be seen in **Figure 2**, performance for both novel metaphor and novel metonymy was well below that for the TD controls suggesting that across the large age span performance in participants with ASD was impaired.

As CA was not a good predictor for performance in the ASD group, the relationship between performance on novel M&M comprehension and verbal abilities as measured by BPVS for younger participants and Vocabulary scale from WASI for adults, and the relationship between performance and non-verbal ability scores from PC for younger participants and Block building from WASI for adults were examined. As shown in **Table 1**, the younger ASD participants did not differ from the control groups for vocabulary comprehension or visuo-spatial construction abilities (all *p*s > 0.05). In contrast, the adults with ASD scored significantly below their age-matched control group for both verbal and non-verbal ability (all *p*s < 0.05; see **Table 1**). These differences will be further explored in Section “Discussion.”

**Table 1:** Participants details for Experiment 1: participants’ ages, block building, and vocabulary abilities per age group and experimental group.

	Group	ASD			TD			
Task	Age group	*N*	Mean	*SD*	*N*	Mean	*SD*	*t*-Test
Block (T-score)	Children	15	114.75	20.11	18	128.50	28.26	*t*(33) = -1.4046, *p* = 0.303, *d* = 0.349
	Adults	16	25.92	18.51	15	50.83	7.13	*t*(18.529) = -4.532, *p* < 0.001, *d* = 1.61
Vocabulary (raw score)	Children	17	82.83	29.82	18	96.83	24.92	*t*(34) = -0.818, *p* = 0.419, *d* = 0.273
	Adults	16	25.54	11.16	16	40.38	4.29	*t*(20.526) = -5.177, *p* < 0.001, *d* = 1.83

In the younger group with ASD, there was a negative correlation between performance on novel metonymy and visuo-spatial abilities: *r*(17) = -0.470, *p* = 0.029. In adults with ASD, on the other hand, performance on novel metaphor comprehension was positively correlated with visuo-spatial abilities: *r*(16) = 0.570, *p* = 0.011, while novel metonymy comprehension correlated positively with vocabulary scores: *r*(16) = 0.491, *p* = 0.027. All other correlations were not significant.

### Discussion

Experiment 1 examined the development of novel metaphor and metonymy comprehension by means of a cross-sectional sample of participants with ASD. At first, we only included children and adolescents in the sample. However, as performance was well below that of CA-matched controls, we expanded the sample to adults as well. Overall, chronological age was not a good predictor for metaphor and metonymy comprehension in participants with ASD in contrast to the TD group. Although previous studies have shown that CA is not a good predictor for lexicalized metaphor comprehension (see discussion in Gernsbacher and Pripas-Kapit, 2012 and meta-analysis by Kalandadze et al., 2016), the current study is the first to show that CA is also not a good predictor for novel metaphor and metonymy comprehension in ASD. In addition, the results showed that performance on both novel metaphor and metonymy was below that of TD controls across the age span included.

For the younger participants, the current results are in line with Olofson et al. (2014) suggesting an impairment in novel metaphor comprehension. There are a number of reasons why our results might differ from Zheng et al. (2015). Kövecses (2010) has shown that there are cultural variations in the use of metaphors across different cultures and thus, it may be that the use of metaphors, and specifically the use of metaphors with the format ‘x is y,’ is more prevalent in Chinese than in English which may impact on familiarity and comprehension proficiency of these expressions in Chinese children with ASD.

The current findings are also in contrast with previous studies that examined comprehension of novel metaphor in adults with ASD and did not show any differences between the ASD and control groups (Hermann et al., 2013; Kasirer and Mashal, 2014). One possible explanation for the discrepancy in outcomes is that the adult participants in the current study showed poorer language and non-verbal abilities, in contrast to adults included in previous studies. Similar to previously argued, weaker figurative language abilities in individuals with ASD might be caused by language difficulties (see Gernsbacher and Pripas-Kapit, 2012, for a discussion) and weaker mental flexibility (Kasirer and Mashal, 2014). We, therefore, examined whether non-verbal abilities or vocabulary scores were good predictors for novel metaphor and metonymy comprehension in our ASD sample.

For ASD adults, we found a positive correlation between non-verbal ability and novel metaphor comprehension. While atypical visuo-spatial processing is well-established for individuals with ASD (McGrath et al., 2012), the underlying reasons for this ability and, in turn, its potential effect on figurative language processing remain to be fully explored. McGrath and colleagues suggested that TD and ASD participants may be tapping into qualitatively different visuo-spatial mechanisms. Previous studies have highlighted a disadvantage of a strong preference for local processing on metaphor comprehension (Happé and Frith, 2006). Recently, Perreault et al. (2011) hypothesized that ASD individuals with superior performance on visuo-spatial tasks can parallel process both local and global information. Hence, we should not automatically equate a high visuo-spatial score with local processing. A dual processing ability could explain the positive correlation found here between non-verbal performance and novel metaphor comprehension in adults with ASD. Studies specifically targeting local versus global processing in children with ASD have found no evidence for dual processing in this age group (Koldewyn et al., 2013), and the study by Perreault and colleagues only tested ASD individuals aged 14–35 years. If dual processing in ASD is acquired later in life, this would explain why only our adult group displayed a positive association. Further work on dual processing and incorporation of visuo-spatial tasks, such as those used by Perreault and colleagues, in figurative language studies are needed to confirm our conclusions here.

Examination of what cognitive abilities are correlated to novel metonymy performance showed that for younger participants those who had higher visuo-spatial abilities performed worse on novel metonymy comprehension, while no such relationship was found for adults. This result is similar to Rundblad and Annaz (2010b) who found that children with ASD with higher scores on the PC task understood fewer lexicalized metonyms. As discussed above, it is unlikely that ASD individuals can tap into global processing through a dual processing ability in childhood. Instead, high scores on the PC task are most likely indicative of younger participants with a strong bias toward local processing, which in turn would hamper their metonymy comprehension. The lack of a correlation for adults is most likely due to the fact that metonyms are cognitively simpler to process than metaphors (Rundblad and Annaz, 2010a), therefore do not require a superior non-verbal ability to ensure better comprehension.

Turning to the impact of vocabulary ability, Kasirer and Mashal (2014) have argued that novel expressions require the creation of novel associations, which could suggest no link between verbal ability and comprehension of novel figurative language expressions. When we tested this, no relationship was found between verbal abilities and novel metaphor comprehension in either the younger or the adult ASD groups, supporting previous studies (Kasirer and Mashal, 2014; Olofson et al., 2014). However, this finding is different from Zheng et al. (2015) who found that, in Chinese children with High Functioning ASD, performance on novel metaphors was related to vocabulary comprehension scores (see earlier discussion of Chinese metaphors).

With regards to novel metonymy, we found that comprehension of novel metonyms was better in those adults with better verbal abilities. There are currently no other studies that have examined novel metonymy comprehension in adults and thus it is not possible to compare the results with those from previous studies. As metonymy comprehension was found to be delayed in all ASD participants, this could indicate that performance relies on advanced semantic knowledge, a verbal ability that improves through increasing exposure. In short, the more lexical items a person is exposed to, the better that person becomes as categorizing and linking those and other expressions as well as their meanings. However, we did not find a positive correlation for our younger participants. This discrepancy is most likely a direct result of the two different background tests. While BPVS tests receptive vocabulary, the Vocabulary test from the WASI tests expressive vocabulary. Individuals with ASD have been found to differ in their performance for receptive versus expressive language skills, although which yields the better performance and the magnitude of this difference is still a matter of debate (Kwok et al., 2015). The use of significantly different vocabulary tests was certainly a limitation of the current study. Originally, we intended to only examine performance on novel metaphors and metonyms in young participants with ASD as most studies had shown that comprehension in adults was not impaired (see for example Kasirer and Mashal, 2014). Yet, as it became clear that our adolescent participants with ASD did not reach ceiling levels on the task, adult participants with ASD were included in the study requiring administration of different cognitive ability tasks.

Cross-sectional studies in atypical populations examine the relationship between performance and mental abilities, through a large number of “snapshots” taken from different individuals of different ages. The individual differences between these participants can mask certain developmental trajectories and relationships (see for example Cornish et al., 2013, for a discussion). Indeed, it has been shown that ASD is very heterogeneous with a lot of variability within the etiology and phenotypic presentation of people with ASD (see Charman, 2015, for a discussion). Thus, variability may have affected the study’s outcomes. Examination of the cognitive profiles of the participants with ASD included in this study show a number of differences between the younger participant group and the adult one. For example, the adult group performed significantly lower on both the verbal and non-verbal tasks compared to TD controls, whilst differences observed between the two younger groups were less marked. Thus, our younger and adult groups are different despite sharing the diagnosis of ASD and the adult group seems to include lower functioning individuals with ASD. One possible explanation that the adult group were recruited from local disabilities services and this may have biased the sample to lower functioning individuals with ASD.

Therefore, in order to get a true insight into the development of novel metaphor and metonymy comprehension in ASD, longitudinal studies are required, where the same participants are assessed more than once on the exact same tasks.

## Experiment 2

The results from Experiment 1 suggest that metaphor and metonymy comprehension are impaired in the ASD group in contrast to the TD control group. As variability within a group can skew the true relationship between variables and performance on a task, we re-tested some of the younger participants with ASD to examine the development of novel M&M comprehension, longitudinally. The purpose of Experiment 2 was to see if a longitudinal design would replicate the cross-sectional results of Experiment 1, which had shown no improvement in the ASD participants with age. The TD participants, in contrast, did clearly improve with age; for this reason, and because it was exceedingly difficult to re-recruit the TD participants, Experiment 2 was limited to the younger ASD group.

### Method

#### Participants

Contact details were no longer accurate for eight of the 18 younger ASD participants and two declined to take part again, leaving eight participants with ASD (six males and two females) being re-tested. The time during the first and second testing session varied between 15 and 51 months with an average of 33.50 months (*SD* = 14.93). The participants with ASD who were included in Experiment 2 did not differ from the larger group of young participants with ASD in Experiment 1 for CA, vocabulary comprehension or visuo-spatial abilities (all *p*s > 0.05). Details for these participants at Time 1 and Time 2 can be found in **Table 2**.

**Table 2:** Participants’ details and performance on novel M&M task for those young participants with ASD who were assessed longitudinally in Experiment 2.

	Time 1		Time 2	
	Mean	*SD*	Mean	*SD*
Chronological age (years)	9.34	2.45	12.14	3.11
Block (T-score)	124.67	18.49	165.33	18.17
Vocabulary (raw-score)	92.86	19.57	100.63	20.16
Metaphor	26.84	22.21	71.67	32.02
Metonymy	21.65	27.45	71.89	33.02

#### Materials, Procedure, and Scoring

The background measures and experimental tasks, procedure, and scoring were the same as those described for Experiment 1 above.

### Results

**FIGURE 3:**
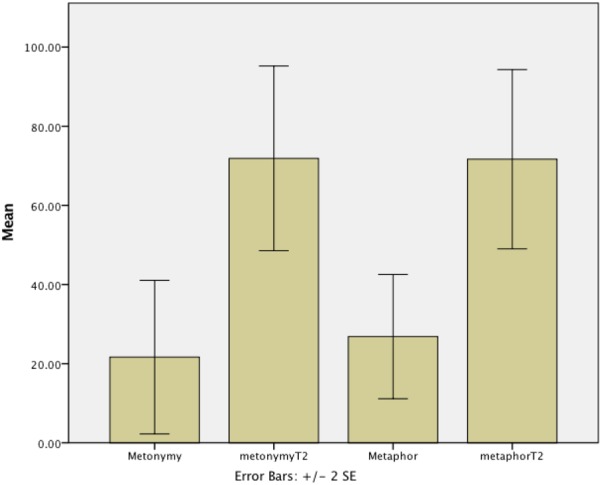
Novel metaphor and novel metonymy comprehension (percentage correct) at Time 1 and Time 2 (T2).

As the time between Experiment 1 and Experiment 2 varied between participants, repeated measures analyses with Time and Type of expression as within factors and Time Difference as a scalar covariate were carried out. There was a significant effect for Time; *F*(1,6) = 11.487, *p* = 0.015, $\eta_{p}^{2}$ = 0.657, and a significant interaction for Time Difference^∗^Time; *F*(1,6) = 93.983, *p* < 0.001, $\eta_{p}^{2}$ = 0.940. There was no effect for Type of expression; *F*(1,6) = 0.012, *p* = 0.915, $\eta_{p}^{2}$ = 0.002. This shows that scores improved similarly for novel metaphors and metonyms and that those participants who had a larger time gap between the two assessments saw a greater improvement. **Figure 3** displays the overall scores for metaphor and metonymy comprehension at Time 1 and Time 2.

### Discussion

This study is the first to examine the development of novel metaphor and metonymy comprehension in children and adolescents with ASD longitudinally, in order to examine whether comprehension scores increase over time. It was found that performance scores for novel metonyms and metaphors increased in participants with ASD as they get older. There were no overall differences between novel metaphor or metonymy comprehension at Time 2. For both types of expression, performance increased, confirming the results for Experiment 1, and the greater the time gap between experiments, the greater the improvement.

Although the results included large effect sizes, one limitation of the current longitudinal study is that only a small sample of children were available for re-testing. In addition, no longitudinal data was included from TD controls and thus it is unclear whether the observed improvement with time in the ASD sub-group is beyond typical improvement with time or the effect of them being tested twice. Thus, these results should be replicated in further studies with a larger and more diverse participant sample.

## General Discussion

This study examined comprehension of novel metaphor and metonymy in young individuals and adults with ASD and is the first, to our knowledge, to compare performance using both a cross-sectional as well as a longitudinal design. Firstly and most importantly, the outcomes of these experiments showed that, although CA was not a good predictor for figurative language comprehension abilities using a cross-sectional design, the results from the longitudinal study showed that participants’ figurative language abilities improved as they got older. Secondly, comprehension in both younger as well as older participants with ASD was consistently lower compared to TD controls. These findings are in line with previous studies that have examined primary conceptual metaphors (Olofson et al., 2014) and lexicalized metonyms (Rundblad and Annaz, 2010a).

Further, Experiment 1 examined the relationship between novel metaphor and metonymy comprehension and other cognitive abilities, including verbal abilities and visuo-spatial abilities. Whilst performance in the adults with ASD correlated with verbal abilities for novel metonymy and with visuo-spatial abilities for novel metaphor comprehension, none of the cognitive abilities related positively to overall performance in the young participants with ASD. These findings are different from Zheng et al. (2015) who found that in Chinese children with High Functioning ASD, performance on novel metaphors, but not novel metonyms related to vocabulary comprehension scores. One explanation for these different correlational outcomes is the cultural differences in the use and cultural context of the figurative expressions (Kövecses, 2010), as linguistic differences in how people use metaphors and other figurative expressions such as metonyms in a cultural context can affect comprehension and the load on other cognitive abilities, to derive a correct interpretation. Importantly, our results suggest that global processing abilities might be related to the development of novel metaphor comprehension in ASD. However, as only a limited amount of cognitive abilities were included in this study and the fact that children and adults completed different cognitive tasks for vocabulary and visuo-spatial abilities, future studies are required with larger sample sizes to further examine the impact of internal factors (e.g., overall ASD severity, overall cognitive ability, flexibility) as well as external factors (e.g., type of speech and language therapy received, type of education attended) for figurative language comprehension in ASD. Importantly, future studies should include cognitive background measures that span a wide age range such as WASI.

Finally, the findings from both studies showed that caution is required when interpreting results from cross-sectional studies, in that while Experiment 1 showed that age was not a good predictor for performance on either novel metaphor or metonymy comprehension, data from the longitudinal experiment showed that performance improved over time. This study, therefore, questions the outcomes of studies that have only examined performance at one particular point in time and that do not take into account longitudinal aspects or how performance changes over time.

## Conclusion

The current study examined comprehension of novel metaphor and metonymy in participants with ASD across a wide age range, using both a cross-sectional and longitudinal design. The results showed that performance in ASD increases over time. These findings have positive implications for education and intervention studies as they suggest that performance can be improved. However, further studies about the cognitive mechanisms that drive this development are required.

## Ethics Statement

All procedures performed in studies involving human participants were in accordance with the ethical standards of the institutional and/or national research committee and with the 1964 Helsinki declaration and its later amendments or comparable ethical standards.

## Author Contributions

JVH and GR both designed the study and stimuli and wrote the manuscript. JVH oversaw the data collection and analyzed the data.

## Conflict of Interest Statement

The authors declare that the research was conducted in the absence of any commercial or financial relationships that could be construed as a potential conflict of interest.
